# A novel, cryopreserved, viable osteochondral allograft designed to augment marrow stimulation for articular cartilage repair

**DOI:** 10.1186/s13018-015-0209-5

**Published:** 2015-05-14

**Authors:** Sandra Geraghty, Jin-Qiang Kuang, Dana Yoo, Michelle LeRoux-Williams, C. Thomas Vangsness, Alla Danilkovitch

**Affiliations:** Osiris Therapeutics, Inc., Albert Einstein Drive, Columbia, MD USA; Tissue Banks International, Park Avenue, Baltimore, MD USA; Department of Orthopaedics, Keck School of Medicine, University of Southern California, San Pablo Street, Los Angeles, CA USA

**Keywords:** Osteochondral allograft, Articular cartilage repair, Marrow stimulation, Microfracture, Chondrocytes, Cryopreservation, *In vitro*, *In vivo*

## Abstract

**Background:**

Here, we describe the design and characterization of a novel, cryopreserved, viable osteochondral allograft (CVOCA), along with evidence that the CVOCA can improve outcomes of marrow stimulation for articular cartilage repair.

**Methods:**

Histological staining was performed to evaluate the CVOCA tissue architecture. CVOCAs were tested for the presence of extracellular matrix (ECM) proteins and chondrogenic growth factors using ELISA. Cell viability and composition were examined via live/dead staining, fluorescence-activated cell sorting (FACS) analysis, and immunofluorescence staining. FACS analysis and a TNF-α secretion bioassay were used to confirm the lack of immunogenic cells. Effects of the CVOCA on mesenchymal stem cells (MSCs) were tested using *in vitro* migration and chondrogenesis assays. The ability of the CVOCA to augment marrow stimulation *in vivo* was evaluated in a goat model.

**Results:**

A method of tissue processing and preservation was developed resulting in a CVOCA with pores and minimal bone. The pores were found to increase the flexibility of the CVOCA and enhance growth factor release. Histological staining revealed that all three zones of hyaline cartilage were preserved within the CVOCA. Chondrogenic growth factors (TGF-β1, TGF-β3, BMP-2, BMP-4, BMP-7, bFGF, IGF-1) and ECM proteins (type II collagen, hyaluronan) were retained within the CVOCA, and their sustained release in culture was observed (TGF β1, TGF-β2, aggrecan). The cells within the CVOCA were confirmed to be chondrocytes and remained viable and functional post-thaw. Immunogenicity testing confirmed the absence of immunogenic cells. The CVOCA induced MSC migration and chondrogenesis *in vitro*. Experimental results using devitalized flash frozen osteochondral allografts revealed the importance of preserving all components of articular cartilage in the CVOCA. Goats treated with the CVOCA and marrow stimulation exhibited better repair compared to goats treated with marrow stimulation alone.

**Conclusions:**

The CVOCA retains viable chondrocytes, chondrogenic growth factors, and ECM proteins within the intact architecture of native hyaline cartilage. The CVOCA promotes MSC migration and chondrogenesis following marrow stimulation, improving articular cartilage repair.

## Background

Articular cartilage damage is a common orthopedic problem, with more than 60 % of knee arthroscopies revealing chondral or osteochondral defects [1, 2]. The capacity for spontaneous articular cartilage repair is very limited [3–6]. Therefore, several surgical options have emerged to reduce pain and restore the structure and function of injured articular cartilage. Marrow stimulation, a reparative approach, and osteochondral allografts, a restorative approach, are commonly employed techniques.

Marrow stimulation is often employed following debridement to fill a lesion with repair tissue [5, 7]. In marrow stimulation, the subchondral bone is penetrated in order to introduce MSCs from the bone marrow into the lesion to generate repair tissue. The initial blood clot filling the lesion typically becomes fibrocartilage repair tissue, with less type II collagen and reduced mechanical integrity compared to normal, hyaline cartilage [8–10]. While marrow stimulation generally has good results in young patients (<40 years old) with small lesions (<2 cm^2^), the fibrocartilage often breaks down over time leading to poor long-term outcomes, especially in older patients and in lesions larger than 2 cm^2^ [8, 10–14]. Several adjuncts to marrow stimulation have recently been developed with the goal of directing the MSCs to become chondrocytes that produce hyaline cartilage. Various scaffolds, cells, and growth factors have been studied for this purpose, but their clinical benefits are not yet clearly defined.

Osteochondral allografting is a restorative procedure in which a cadaveric graft consisting of articular cartilage and its underlying subchondral bone is transplanted into the defect. Osteochondral allografts can be used to treat larger osteochondral lesions, but the procedure relies on precise instrumentation to match the size and contours of the donor tissue to the patient lesion. The clinical use of fresh and fresh stored osteochondral allografts began more than 40 years ago and has shown good results [7, 15]. As the implantation technique and selection criteria have improved, long-term clinical success rates have increased to 80–85 % on average, and above 95 % in more recent studies of knee defect repair [16–20]. Currently, fresh osteochondral allografts are stored at 4 °C in media for approximately 14 days while microbiological screening and recipient matching is completed and are typically used within 30 days, the time point when chondrocyte viability begins to decline significantly [21–24]. The short shelf life and size-matched donor availability limit the use of fresh stored osteochondral allografts. Frozen osteochondral allografts offer a longer shelf life, but they lose chondrocyte viability and have a higher likelihood of delaminating from the surrounding tissue [25, 26].

Here, we present a new option that combines reparative and restorative approaches for articular cartilage repair. The cryopreserved, viable osteochondral allograft (CVOCA) is a novel osteochondral allograft with only a minimal amount of bone and pores spanning the thickness of the allograft. The CVOCA builds upon more than 40 years of safety and efficacy of fresh stored osteochondral allografts as a restorative approach to articular cartilage repair. In contrast to fresh stored osteochondral allografts, the CVOCA can be stored frozen for up to 2 years, can fit to any surface contour, and can be implanted arthroscopically. In addition to increasing the flexibility of the CVOCA, the pores enable the cryopreservation solution to penetrate the tissue to preserve cell viability throughout rather than just at the surface, as seen in previous attempts at cartilage cryopreservation [27]. When the CVOCA is used with marrow stimulation, MSCs released from the bone marrow will initiate a reparative response within the lesion in addition to the restorative approach from the allograft cartilage itself. The MSCs will be stimulated to undergo chondrogenesis within the pores of the CVOCA and produce type II collagen-rich hyaline repair tissue. In this study, we characterize the CVOCA and provide data supporting our hypothesis that the novel CVOCA can improve articular cartilage repair when used with marrow stimulation.

## Methods

### Tissue procurement and CVOCA preparation

Human knees en bloc from 18 to 60-year-old donors that were deemed as eligible for tissue donation for medical research were shipped on wet ice and processed within 120 h post-mortem. Osteochondral plugs (20 mm and 10 mm in diameter, 1 to 3 mm thick) were harvested from regions of healthy cartilage in the distal femur and proximal tibia. Most of the osseous portion of each plug was removed, leaving osteochondral allografts retaining the entire thickness of hyaline cartilage and residual subchondral bone. A score mark was added to the bone side of each osteochondral allograft to maintain directionality. Pores spanning the full thickness of the osteochondral allografts were created. The CVOCAs were cryopreserved in a DMSO-based solution using a controlled rate freezer (Cryomed Model 7454) and stored at —80 °C. When ready for testing, the CVOCAs were thawed in a 37 °C water bath and rinsed in saline.

In designing the CVOCA, the importance of the pores and their optimum size and density were investigated. Pore diameters of 0.6 and 0.9 mm and densities of 12, 25, and 50 pores/cm^2^ were tested for flexibility. Six blinded evaluators rated the flexibility of each CVOCA on a scale from 1 to 5 (1 = most flexible, 5 = least flexible). TGF-β1 release from the CVOCA (10-mm diameter size) with pores (1-mm diameter, 36 pores/cm^2^) and without pores after a 7 day culture (in Dulbecco's modified Eagle's medium (DMEM) with 10 % fetal bovine serum (FBS), 2 % Glutamax, 2 % antibiotic-antimycotic) was measured by ELISA (TGF-β1 Quantikine kit, R&D Systems, Minneapolis, MN, USA).

### Histological characterization and immunostaining of CVOCA

The structure and extracellular matrix (ECM) contents of the CVOCA were investigated through histological staining of vertical cross sections. CVOCAs were fixed in 4 % paraformaldehyde, decalcified in 10 % formic acid, and embedded in paraffin. Serial 5-μm thick sections from the center of the CVOCA were stained with hematoxylin and eosin (H&E), safranin O, and antibodies for type I and type II collagen according to standard procedures. In negative control sections, normal rabbit serum was substituted for the primary antibody. Positive control sections included sections of normal human articular cartilage (for type II collagen) and subchondral bone (for type I collagen).

### ELISA for ECM proteins in CVOCA

CVOCA tissue lysates and culture supernatants were analyzed using ELISAs for the presence of ECM proteins. CVOCAs were homogenized and the resulting tissue lysates were analyzed using ELISA kits for type II collagen (Astarte Biologics, Bothell, WA, USA) and hyaluronan (R&D Systems, Minneapolis, MN, USA) following the manufacturer’s protocols. CVOCAs were cultured with chondrocyte growth culture media (Lonza, Basel, Switzerland) at 37 °C and 5 % CO_2_. Media collected after 1, 3, 7, and 14 days was analyzed using an aggrecan ELISA kit (DIAsource ImmunoAssays, Louvain-la-Neuve, Belgium) following the manufacturer’s protocol.

### ELISA for chondrogenic growth factors in CVOCA

Chondrogenic growth factors were evaluated in CVOCA tissue lysates using Quantikine and DuoSet ELISA kits (R&D Systems, Minneapolis, MN, USA) for TGF-β1, TGF-β3, BMP-2, BMP-4, BMP-7, bFGF, and IGF-1 following the manufacturer’s protocols. Culture supernatants were collected after 7, 14, and 21 days of CVOCA culture at 37 °C and 5 % CO_2_ in chondrocyte growth culture media (Lonza, Basel, Switzerland). The culture supernatants were analyzed using DuoSet ELISA kits (R&D Systems, Minneapolis, MN, USA) for TGF-β1, TGF-β2, bFGF, and IGF-1 following the manufacturer’s protocols. For comparison, the cells were devitalized in some samples by freeze-thawing the CVOCAs three consecutive times in a liquid nitrogen bath to create flash frozen OCAs before use in the culture.

### Live/dead cell staining in CVOCA

Cell distribution and viability within the CVOCA were investigated using live/dead cell staining. A scalpel blade was used to cut thin horizontal tissue sections from the superficial end of the CVOCAs. The sections were incubated with a live/dead viability/cytotoxicity staining solution (Molecular Probes, Inc., Eugene, OR, USA) according to the manufacturer’s instructions. Stained sections were photographed under 100× magnification using a fluorescent microscope. Viable (green) and dead (red) cells within three random fields were counted within each of the CVOCA tissue sections. The counts from the three fields were averaged, and the percentage of viable cells was calculated for each CVOCA.

### Isolation and analysis of cells from CVOCA

Cells were isolated by incubating minced CVOCAs with a pronase digestion media (70 U/ml, Sigma, St. Louis, Missouri, USA) for 1 h followed by a type I collagenase digestion media (300 U/ml, Sigma, St. Louis, Missouri, USA) for 3 h. Isolated cells were cultured in growth media (DMEM/Ham’s F-12 with 10 % FBS, 50 μg/ml ascorbic acid, 1 mg/ml glucose, and 0.2 % gentamicin) and passaged three to five times.

The cellular composition within the CVOCA was investigated through fluorescence-activated cell sorting (FACS). Cell suspensions were stained with phycoerythrin (PE)-labelled antibodies to CD44, CD45, CD49e, IgG1 (isotype control), or IgG2bκ (isotype control) (BD Biosciences, San Jose, CA, USA) according to standard procedures. The stained cells were evaluated using a single color analysis on a FACSCalibur system with CellQuest software (BD Biosciences, San Jose, CA, USA).

The cells isolated from the CVOCA were further characterized by immunostaining in culture for type II collagen and aggrecan according to standard protocols, with rabbit polyclonal anti-type II collagen and mouse monoclonal anti-aggrecan (Abcam, Cambridge, UK) used as the primary antibodies and Alexa Fluor 594 donkey anti-rabbit and Alexa Fluor 488 rabbit anti-mouse (Invitrogen, Grand Island, NY, USA) used as the secondary antibodies. Primary antibodies were not included into staining for negative control samples. All samples were stained with DAPI (Roche, Basel, Switzerland) and analyzed with a fluorescence microscope.

### LPS-induced TNF-α secretion assay

Raw material (10-mm diameter osteochondral plugs with approximately 5 mm of bone) and fully processed CVOCAs (10-mm diameter) sourced from the same donor were cultured within wells of a 24-well plate in 2 ml of chondrocyte growth culture media (Lonza, Basel, Switzerland) per well. The samples were exposed to bacterial lipopolysaccharide (LPS) (1 μg/ml, Sigma, St. Louis, Missouri, USA) for 24 h at 37 °C. Human peripheral blood mononuclear cells (hPBMCs) cultured with LPS (2 × 10^6^ cells/well) served as a positive control. Samples without LPS served as background controls. After the 24-h culture period, the media was collected and tested for the presence of TNF-α using a TNF-α DuoSet ELISA kit (R&D Systems, Minneapolis, MN, USA) according to the manufacturer’s protocol.

### *In vitro* mesenchymal stem cell migration assay

A transwell filter system was utilized to measure the migration of MSCs towards media conditioned with CVOCAs or flash frozen OCAs. The CVOCAs and flash frozen OCAs (20-mm diameter) were cultured at 37 °C and 5 % CO_2_ in 3 ml of media (DMEM + 2 % antibiotic-antimycotic) for 7 days to create the conditioned media. DMEM was used as the negative control media while DMEM + 10 % FBS served as the positive control media. Six hundred microliters of the media was placed within wells of a 24-well plate. A 0.33-cm^2^ filter with 8-μm pores was added to each well so the media filled the well up to the filter. 1 × 10^5^ human bone marrow-derived MSCs at passage 3–5 were seeded on the top of each filter. After a 4-h culture, the MSCs that did not migrate across the filters were removed from the top of each filter using cotton buds. The migrated MSCs were fixed and stained with a 4 % paraformaldehyde/1 % gentian violet solution. The migrated cells were visualized using light microscopy. The migrated cells were then removed from the bottom of each filter using 0.25 % trypsin-EDTA, spun down, resuspended in DMEM, and counted using a hemocytometer.

### *In vitro* mesenchymal stem cell chondrogenesis assays

The ability of the CVOCA to induce MSC chondrogenesis was investigated using two *in vitro* CVOCA and MSC co-culture assays. First, MSC pellets were co-cultured with CVOCAs, but the two were not in direct contact. 2.5 × 10^5^ human bone marrow-derived MSCs at passage 3–5 were pelleted into 15-ml conical tubes. A basal media (incomplete chondrogenic induction media, Lonza, Basel, Switzerland) was added to each tube. The basal media alone served as the negative control while the CVOCA (1/4 of 20-mm diameter CVOCA) was added to each experimental tube so it resided within the media but above the cell pellet for co-culture within the conical tube. After 3 weeks of culture, the pellets were sectioned and stained for type II collagen, using the standard protocol. The percentage of each pellet cross section that stained positive for type II collagen was calculated using ImageJ (US National Institutes of Health, Bethesda, Maryland, USA).

In order to mimic the *in vivo* microenvironment following microfracture and implantation of the CVOCA, a second *in vitro* chondrogenesis assay was performed where MSCs were suspended in fibrin glue and spread to fill the pores in the CVOCA with MSCs. Some of the CVOCAs were flash frozen in liquid nitrogen, as described previously. Each OCA (20-mm diameter) was placed in a well within a 24-well plate and 4 × 10^6^ human bone marrow-derived MSCs at passage 3–5 in 100 μl of fibrin glue were spread over the CVOCA. One milliliter of complete or incomplete chondrogenic induction media (Lonza, Basel, Switzerland) was added to each well. After 4 weeks, the samples were sectioned vertically and horizontally and stained for type II collagen. The percentage of pore cross sections that stained positive for type II collagen was calculated using ImageJ (US National Institutes of Health, Bethesda, Maryland, USA).

### CVOCA testing in a goat microfracture model

The CVOCAs were implanted in goats in order to demonstrate safety and to investigate the time course of integration following implantation. Approval was obtained from the Institutional Animal Care and Use Committee (IACUC) of Thomas D. Morris, Inc. for the use of animals in this study. Young (<1 year old) Spanish goat CVOCAs of 6-mm diameter were prepared using the same methods that were developed for human tissue processing. The goat CVOCAs were implanted in nine skeletally mature (2–4 years old), female Spanish goats. In each goat, two critical size cartilage defects (6 mm in diameter) were created in the medial femoral condyle of the right anterior knee joint using a biopsy punch and cartilage dissection tools. The defects in 3 goats (6 defects) were treated with marrow stimulation alone. The defects in the remaining 6 goats (12 defects) were treated with marrow stimulation augmented with the CVOCA. Following marrow stimulation, the CVOCA was secured in place by one of three methods: fibrin glue below and above the CVOCA (6 defects), fibrin glue above the CVOCA only (2 defects), or four 7-0 Vicryl sutures spaced evenly around the CVOCA and through the surrounding healthy cartilage (4 defects). The goats were fitted with a modified Thomas splint and cast immediately after surgery. The cast was removed after 21 days.

The goats were humanely euthanized after 3 months (6 goats) and 12 months (3 goats) for analysis. Synovial fluid was collected and evaluated for color and viscosity. Popliteal lymph nodes were examined for inflammation. The contralateral joint and the articulating surface opposing the defects were examined for any abnormalities in each goat. An investigator blinded to the treatments examined the operated joints, grading each lesion site using the parameters outlined in Table 1. The sum of the gross morphology scores was multiplied by the estimated total percentage fill to assign each lesion a normalized grade score (maximum possible = 10). Five micron thick sections were taken through the center of the defects as well as through medial and lateral edges of the defects (1.5 cm from center). Serial sections were stained with H&E and safranin O, and immunohistochemistry was performed for type I and type II collagen. All stained sections were imaged and analyzed by two independent, blinded reviewers (SG and JQK) using the Pineda repair grading system [28]. The reviewers’ scores from all three sites (medial edge, center, lateral edge) were averaged to give the overall histology scores for each lesion.

**Table 1:** Repair tissue gross morphology scoring parameters. Parameters used to score goat repair tissue morphology. The total score was multiplied by the total percentage of defect fill to give the normalized grade score for each goat lesion

Characteristic	Grading	Score
Test article or repair tissue presence in defect	Present	2
	Partial	1
	None visible	0
Edge integration (new tissue relative to native cartilage)	Present	2
	Partial	1
	None visible	0
Smoothness of the cartilage surface	Present	2
	Partial	1
	None visible	0
Cartilage surface, degree of filling	Present	2
	Partial	1
	None visible	0
Color of cartilage, opacity, or translucency of the neocartilage	Present	2
	Partial	1
	None visible	0

### Statistical analysis

Each experiment was run on CVOCAs from multiple donors. Data are presented as the mean ± standard error of the mean (SEM). Comparisons between groups were made using Student’s t-tests with significance at *p* < 0.05.

## Results

### CVOCA design

**Fig. 1:**
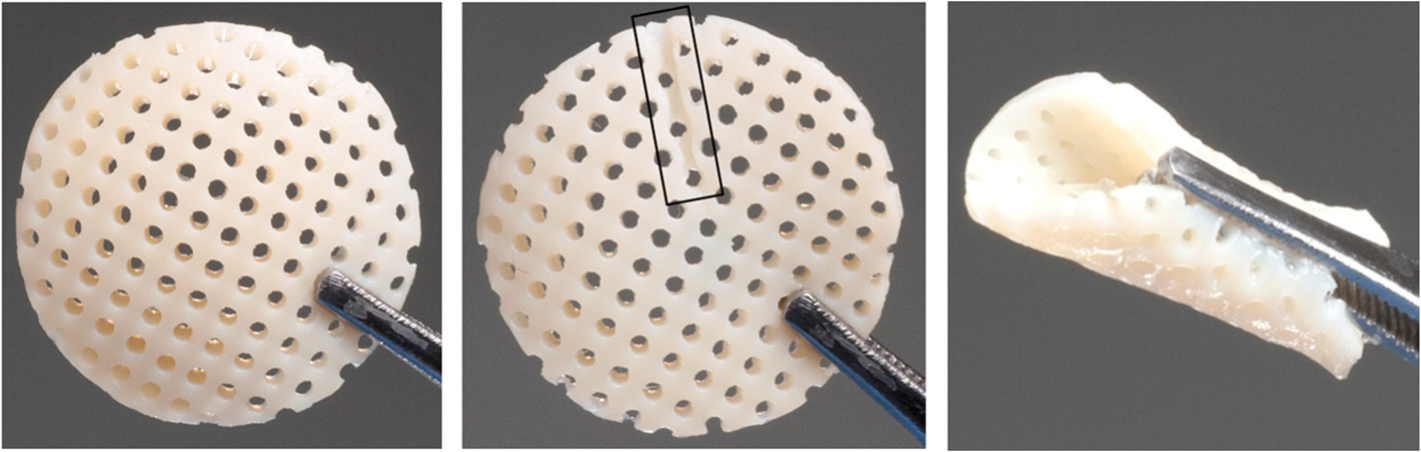
Appearance of CVOCA. Appearance of a CVOCA (20-mm diameter size): top (*left*), bottom (*middle*), and side (*right*) views. Note the score mark distinguishing the bottom (bone) side (*outlined with black box in the middle image*) and the flexibility that enables folding (*right image*)

The main goals in the novel CVOCA design were to preserve all the benefits of a traditional fresh stored osteochondral allograft including maintenance of the articular cartilage structure and high chondrocyte viability, while at the same time making it flexible, easy to implant, compatible with arthroscopy, and able to have a prolonged storage time. Only a minimal amount of bone is kept to minimize damage to the patient’s subchondral bone while still providing an anchor for bony integration. Additionally, reducing the amount of bone in the CVOCA compared to traditional OCAs, along with the added pores, allows the CVOCA to be flexible enough to be implanted arthroscopically. The flexible allograft can be easily sized and adapted to different contours upon implantation (Fig. 1). The pores also enable the cryopreservation solution to penetrate the CVOCA, maintaining chondrocyte viability throughout the CVOCA rather than just at the surface. Additionally, the pores provide a favorable chondrogenic microenvironment for autologous MSCs, which are released from bone marrow into the cartilage defect following a marrow stimulation procedure and will reside within the CVOCA pores. The viable hyaline cartilage within the CVOCA is expected to provide the necessary signals to induce chondrogenesis of MSCs released following marrow stimulation.

The pores were found to increase flexibility of the CVOCA and to enhance growth factor release from the CVOCA. The best flexibility score (2.2 ± 0.3) was assigned to CVOCAs with the large pore size (0.9 mm) at the highest density (50 pores/cm^2^). The worst flexibility score (4.8 ± 0.2) was assigned to CVOCAs with the small pore size (0.6 mm) at the lowest density (12 pores/cm^2^). The CVOCAs with the large pore size (0.9 mm) at the medium density (25 pores/cm^2^) were rated as the second most flexible (3.0 ± 0.4). Based on these results, 1-mm diameter pores at a density of 36 pores/cm^2^ were selected (Fig. 1). Following a 7-day culture, it was observed that CVOCAs with pores released more TGF-β1 than CVOCAs without pores (2,020 vs. 3,371 pg/ml). This boost may be attributed to the approximately 34 % overall increase in surface area that the 1-mm pores at 36 pores/cm^2^ provide.

### Extracellular matrix content

**Fig. 2:**
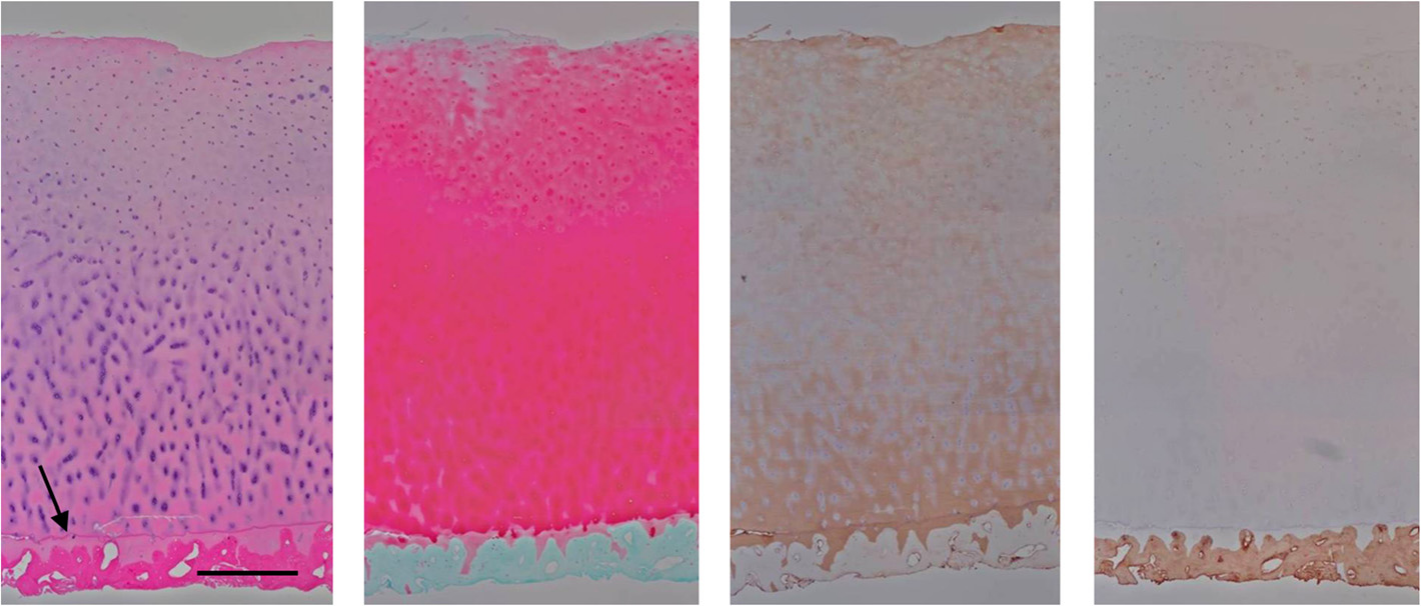
CVOCA maintains hyaline cartilage structure and ECM contents. Histological stains of a representative cross section of the CVOCA show that hyaline cartilage architecture (superficial, transitional, radial zones) and ECM contents (aggrecan, type II collagen) are maintained in the CVOCA. Stains *from left to right*: Hematoxylin & Eosin, Safranin O, Type II Collagen, and Type I Collagen. The *black arrow* points to the tidemark, marking the intersection of the hyaline cartilage and the thin layer of subchondral bone. The scale bar represents 500 μm

**Fig. 3:**
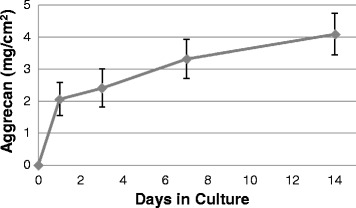
CVOCA releases aggrecan over time. Aggrecan content in media collected from CVOCA culture at various time points. The CVOCA released aggrecan for up to 14 days, which was the longest experimental time point. Data presented as mean ± SEM for four independent experiments

Histological staining of the CVOCA revealed that normal hyaline cartilage architecture and ECM contents were preserved. The CVOCA retained the superficial, transitional, and radial zones of hyaline cartilage and a thin layer of subchondral bone (Fig. 2). The cellular distribution and morphology, evident in the H&E stain, are characteristic of chondrocytes within hyaline cartilage. The visible tidemark revealed that the transition between cartilage and bone remained intact. The strong red safranin O staining indicated the presence of a proteoglycan-rich matrix. The CVOCA also stained strongly for type II collagen. Analysis of CVOCA tissue lysates revealed that the CVOCA contained type II collagen, hyaluronan, and aggrecan. The amount of aggrecan released by the CVOCA into the culture media increased significantly with each time point (*p* < 0.03) (Fig. 3).

### Growth factor content

Many of the growth factors that are important for cartilage function, both in health and repair, were found to be present within the tissue and secreted by the CVOCA. Analysis of the CVOCA tissue lysates revealed that the CVOCA contained TGF-β1 and TGF-β3, two growth factors that promote chondrogenic differentiation of MSCs and regulate type II collagen expression in chondrocytes [29]. The CVOCA tissue lysates contained BMP-2, BMP-4, and BMP-7. These bone morphogenetic proteins are capable of inducing MSC chondrogenesis and stimulating ECM production by chondrocytes [29]. The CVOCA tissue lysates also contained bFGF, a growth factor that stimulates the proliferation of chondrocytes, and IGF-1, a growth factor that induces ECM synthesis in chondrocytes [29].

**Fig. 4:**
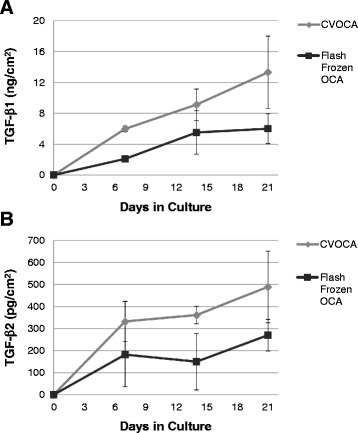
CVOCA releases more TGF-β over time than flash frozen OCA. TGF-β1 (**A**) and TGF-β2 (**B**) content in media collected from CVOCA and flash frozen OCA culture at various time points. The CVOCA releases more of the chondrogenic growth factors initially (day 7) and over time (up to 21 days) relative to the flash frozen OCA. Data presented as mean ± SEM for three independent experiments

Both CVOCAs and flash frozen OCAs released increasing amounts of TGF-β1 into the culture media with time (Fig. 4a). CVOCAs released increasing amounts of TGF-β2 into the culture media with time while flash frozen OCAs followed the same trend except for a slight drop in TGF-β2 release on day 14 (Fig. 4b). The CVOCAs released more TGF-β1 and TGF-β2 than the flash frozen OCAs at every time point. This difference was statistically significant at the day 7 time point for TGF-β1 release (*p* = 0.002).

### Cellular content

**Fig. 5:**
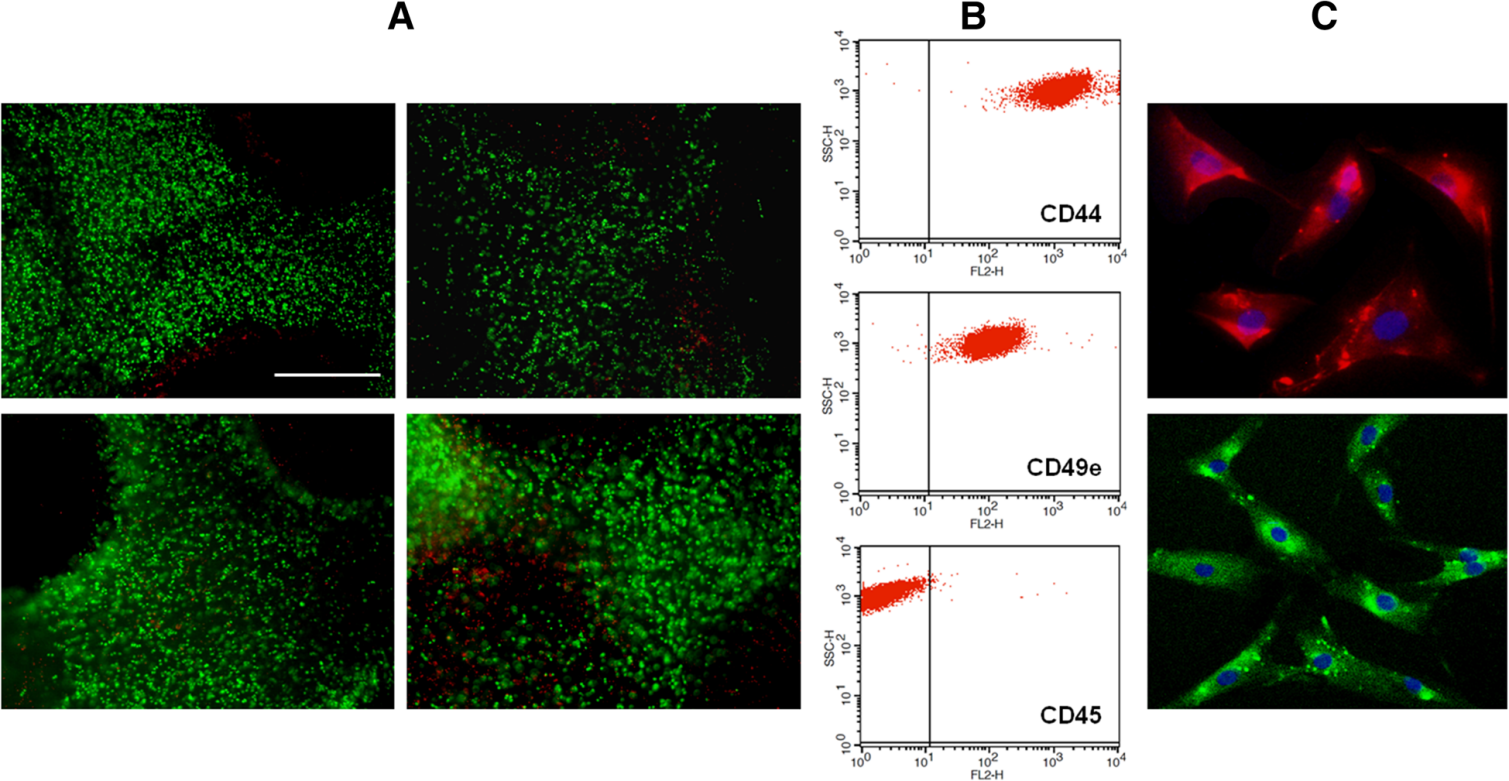
Cells within the CVOCA are viable, functional chondrocytes. (**A**) Representative images of live/dead staining in a transverse section taken from the top (superficial zone) of CVOCAs from four different donors. Live cells are stained *green*, and dead cells are stained *red*. The *scale bar* represents 500 μm. (**B**) Representative FACS *dot* plots for the cell population isolated from the CVOCA. The *vertical lines* on the graphs represent a cut off point for negatively (*left side*) and positively (*right side*) stained cells. The population is positive for the chondrocyte markers CD44 and CD49e and negative for the hematopoietic cell marker CD45. (**C**) Immunofluorescent staining of cultured cells that were isolated from the CVOCA. Cells were stained for nuclei (*blue*, *both images*), type II collagen (*red*, *top image*), and aggrecan (*green*, *bottom image*)

As shown in Fig. 5a, the majority of the viable cells within the CVOCA were retained after cryopreservation. Tissue sections from the superficial zones of the CVOCAs contained 64,989 ± 7665 live cells/cm^2^ with a mean cell viability of 70.5 % (range, 54.5–88.5 %). The percentage of viable cells in CVOCAs post-thaw was not different from the fresh OCA controls from the same donors. The viable cells that were isolated from the CVOCA expressed the hyaluronan receptor CD44 and the fibronectin and fibrinogen receptor CD49e, both chondrocyte markers (Fig. 5b) [30, 31]. The cells were CD45-negative, indicating that the population was free of hematopoietic cells. The cultured cells that were isolated from the CVOCAs stained positively for type II collagen and aggrecan (Fig. 5c), suggesting that the cells within the CVOCA are viable, functioning chondrocytes.

### Immunogenicity

**Fig. 6:**
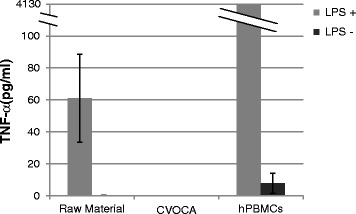
Absence of immunogenic cells in the CVOCA. TNF-α content in media collected following 24-h cultures of freshly isolated osteochondral plugs (raw material), CVOCAs, and hPBMCs (positive control) with and without LPS. TNF-α levels were measured by ELISA. Data presented as mean ± SEM for three independent experiments

The absence of immunogenic CD45-positive hematopoietic cells within the CVOCA was shown by FACS analysis (Fig. 5b). The lack of immunogenicity of the CVOCA was further confirmed in the LPS-induced TNF-α secretion assay. As depicted in Fig. 6, the TNF-α production was negligible when the CVOCA was challenged by LPS (0.04 ± 0.04 pg/ml).

### CVOCA-induced mesenchymal stem cell migration

**Fig. 7:**
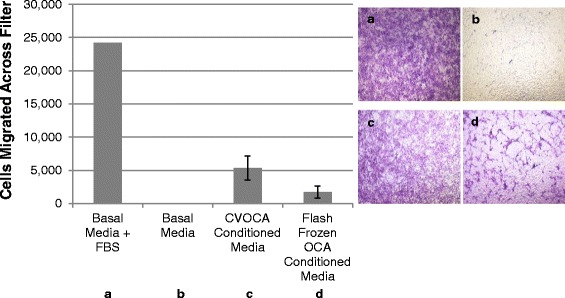
CVOCA recruits MSCs. Total number of MSCs that migrated across porous filters towards different media conditions ((**a**) basal media + FBS, (**b**) basal media, (**c**) COVCA conditioned media, (**d**) flash frozen OCA conditioned media) after 4 h (*bar graph*) along with representative images from each condition showing the migrated MSCs on the *bottom side* of the filters. Data presented as mean ± SEM for three independent experiments

MSCs migrated across porous filters towards CVOCA conditioned media (Fig. 7), indicating that the CVOCA releases factors that are capable of recruiting MSCs. There was a 3.1-fold greater recruitment of MSCs by the CVOCA conditioned media compared to the flash frozen OCA conditioned media. The lower number of migrated MSCs induced by the devitalized flash frozen OCAs was likely due to the reduced growth factor release by the flash frozen OCAs relative to the CVOCAs (Fig. 4).

### CVOCA-induced mesenchymal stem cell chondrogenesis

**Fig. 8:**
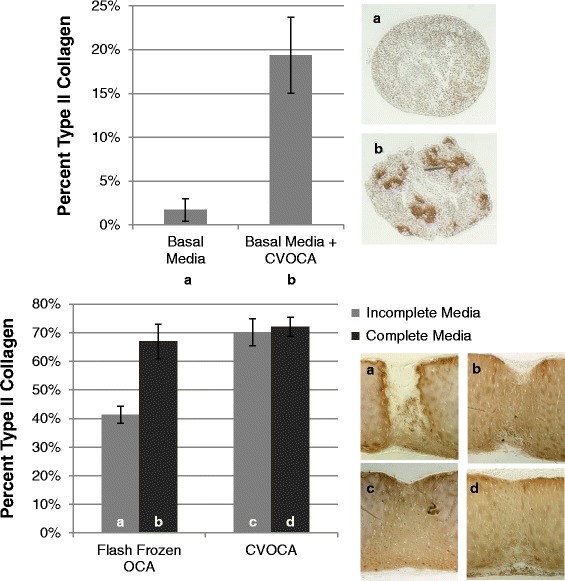
CVOCA induces chondrogenesis of MSCs. Percentage of pellet (*top*) and pore (*bottom*) cross sections that stained positive for type II collagen after culture (pellet—3 weeks, pores—4 weeks) along with representative images of type II collagen staining in pellets and pores (*vertical cross sections*) from each culture condition (*top* – (**a**) basal media, (**b**) basal media + CVOCA, *bottom* – (**a**) flash frozen OCA in incomplete media, (**b**) flash frozen OCA in complete media, (**c**) CVOCA in incomplete media, (**d**) CVOCA in complete media). Data presented as mean ± SEM for two independent experiments

Chondrogenesis, as evidenced by type II collagen production, was observed within MSC populations that were co-cultured with the CVOCA (Fig. 8). 19.3 ± 4.3 % of the MSC population cultured in pellets below the CVOCA for 3 weeks stained positive for type II collagen. In comparison, only 1.7 ± 1.3 % of MSCs in pellets cultured alone were positive for type II collagen. This difference is statistically significant (*p* = 0.02), and these data indicate that the CVOCA releases factors that promote MSC chondrogenesis. When the MSCs were cultured in contact with the CVOCA (within the pores) for 4 weeks, a higher level of MSC chondrogenesis was observed. 72.2 ± 3.3 % of MSCs cultured within pores of a CVOCA with complete chondrogenic induction media were positive for type II collagen (positive control). When the MSCs were cultured within pores of the CVOCA with incomplete chondrogenic induction media (without chondrogenic growth factors), the CVOCAs released a sufficient amount of growth factors, inducing chondrogenesis in 70.1 ± 4.7 % of MSCs. MSCs cultured within pores of the flash frozen OCA demonstrated differentiation (type II collagen staining) in 41.4 ± 3.0 % of the population. This difference between the CVOCAs and the flash frozen OCAs was statistically significant (*p* = 0.02) and highlights the importance of preserving all inherent components of the CVOCA to maintain a high level of chondrogenic potential.

### CVOCA facilitated cartilage repair in a goat microfracture model

All goats remained clinically healthy and showed no local or systemic effects after the surgery. Upon examination after sacrifice, there was no evidence of infection or inflammation and synovial fluid appeared normal in all animals. Any degenerative changes between the operated and contralateral knee were generally matched for the medial femoral condyle, condyle groove junction, femoral groove, and proximal tibia and were not unusual for the animal’s age and species [32].

**Fig. 9:**
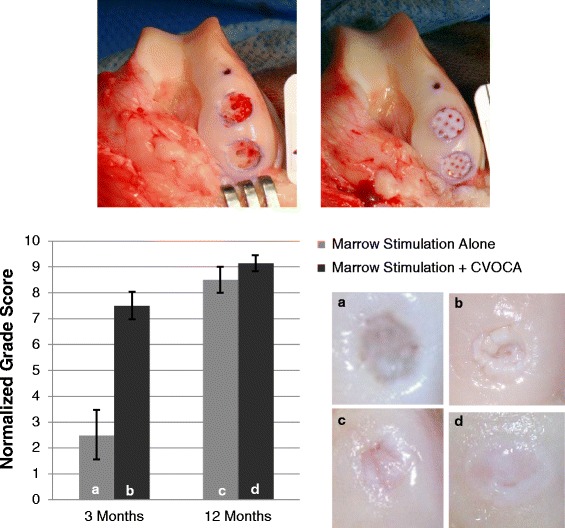
Gross appearance and analysis of lesions in goat model at time of implantation and at 3 and 12 months. Appearance of lesions treated with marrow stimulation alone (*top left*) and marrow stimulation plus implantation of a CVOCA (*top right*) at time of surgical intervention. Normalized grade scores for repair tissue in lesions treated with marrow stimulation alone at 3 months (**a**) and 12 months (**c**) or marrow stimulation plus the CVOCA at 3 months (**b**) and 12 months (**d**), along with representative images of repair tissue in lesions from each condition and time point (*bottom*). The maximum normalized grade score possible is 10. The *bar graph* shows mean ± SEM

As shown in Fig. 9, the normalized grade scores for the lesions treated with marrow stimulation plus the CVOCA were higher than the scores for the lesions treated with marrow stimulation alone, demonstrating that the use of the CVOCA resulted in improved gross morphology. This difference was statistically significant at 3 months when the lesion treated with CVOCA was filled with repair tissue while the lesion treated with marrow stimulation alone was largely unfilled (*p* = 0.006). The normalized grade score for lesions treated with the CVOCA increased significantly from 3 to 12 months as well (*p* = 0.02). By 12 months, boundaries of the CVOCA were no longer distinguishable due to the complete integration of the implanted CVOCA with the repair tissue filling the pores and the surrounding host tissue. This integration is reflected in the maximum possible edge integration score (2.0) within the normalized grade score that the goats treated with the CVOCA received. The lesions treated with marrow stimulation alone had less overall lesion fill at 12 months than the lesions treated with the CVOCA, but the repair tissue appeared normal upon gross analysis and no statistically significant difference in normalized grade scores was observed.

The Pineda histological scoring system addresses four parameters of cartilage repair quality with overall scores ranging from 0 for normal, healthy tissue to 14 for abnormal, damaged tissue [28]. Pineda scores improved significantly for lesions treated with the CVOCA from 6.0 ± 0.8 at 3 months to 2.0 ± 0.6 at 12 months (*p* = 0.003). The reduction in Pineda scores over time was also significant for lesions treated with marrow stimulation alone (7.8 ± 0.7 at 3 months, 3.2 ± 0.2 at 12 months, *p* = 0.006). The scores for defect filling, one of the four parameters that make up the overall Pineda score, were significantly lower for the lesions treated with the CVOCA at both time points (*p* < 0.05). At 3 months, the scores for cell morphology, the measure of hyaline tissue in the Pineda system, were also significantly lower for lesions treated with the CVOCA (*p* = 0.04).

**Fig. 10:**
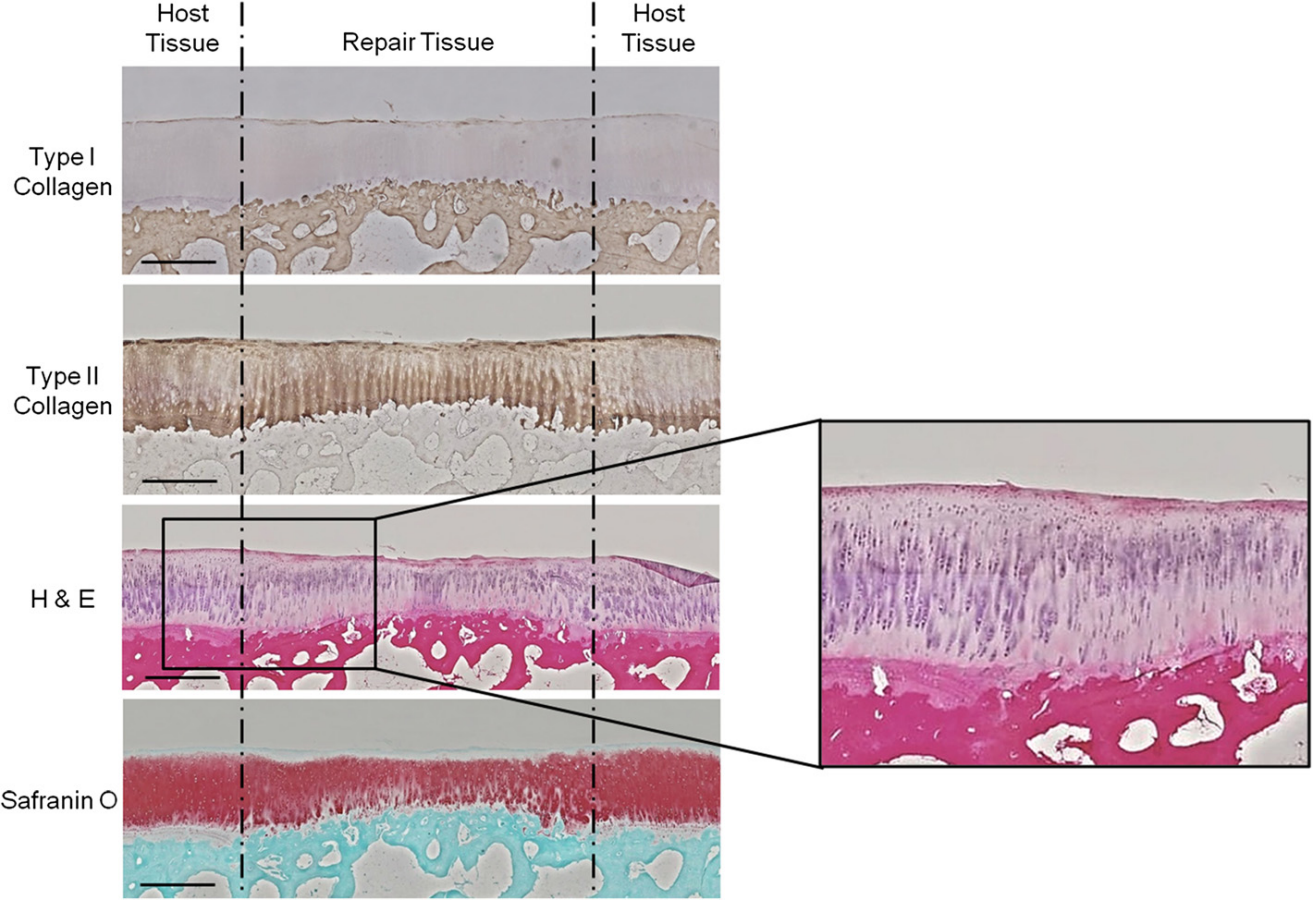
CVOCA repair tissue histology in goat model at 12 months. Representative histological stains of host and repair tissue in lesion treated with marrow stimulation plus the CVOCA at 12 months post-intervention. Sections were taken through the center of the lesion, and the *scale bars* represent 1 mm. The enlarged H&E image shows the border between the host and repair tissues and highlights the cellular organization and tidemark configuration in both tissues

The cartilage lesions treated with the CVOCA were filled with highly cellular, hyaline-like repair tissue at 3 months post-surgery. At both 3 and 12 month time points, the repair tissue contained primarily type II collagen, with a small amount of type I collagen, and the implanted CVOCA completely integrated with the surrounding tissue. By 12 months, aggrecan content increased and cellular morphology and distribution were comparable to the morphology and distribution of cells within normal cartilage, with more rounded cells in the superficial zone and a columnar formation in the transitional and radial zones (Fig. 10). The formation of a new tidemark marked the good integration between the CVOCA implant and the host subchondral bone.

At the 3-month time point, when different methods for implanting the CVOCA were tested, all gross and histological scores were better when either fibrin glue above the CVOCA or sutures were used, rather than fibrin glue below and above the CVOCA. For this reason, it is recommended that the CVOCA is implanted in direct contact with the subchondral bone for clinical use.

## Discussion

In this study, we described the design and characteristics of a novel, cryopreserved, viable osteochondral allograft (CVOCA). Several goals were achieved in designing the CVOCA including flexibility, ease of use, a long storage time, and preservation of the structural matrix, growth factors, and viable chondrocytes. In addition, the CVOCA design allows it to be used in combination with marrow stimulation for articular cartilage repair.

Following a marrow stimulation procedure and implantation of the CVOCA, it is hypothesized that blood containing bone marrow MSCs will fill the pores of the CVOCA, forming stable clots within each pore. The growth factors released by the CVOCA will continue to recruit bone marrow MSCs towards the CVOCA over time. The CVOCA will not only serve as a scaffold for the MSCs, but the viable chondrocytes in the CVOCA will continually release growth factors into the surrounding microenvironment, influencing the MSCs to undergo chondrogenesis and produce hyaline repair tissue within the pores.

Over time, the reparative response initiated within the lesion is expected to not only fill in the pores of the CVOCA but also to integrate the border between the CVOCA and the host cartilage surrounding the defect. The residual bone in the CVOCA will help to anchor the allograft to the host bone through creeping substitution and re-vascularization, similar to the repair seen with traditional fresh stored osteochondral allografts [33, 34]. In this manner, the CVOCA is expected to result in a combination of allogeneic and autologous hyaline cartilage filling the lesion, strongly integrated with surrounding cartilage and underlying bone.

Here, we characterized the structure and contents of the CVOCA and provided *in vitro* and *in vivo* evidence supporting our hypothesis, demonstrating how the CVOCA works to augment marrow stimulation to improve articular cartilage repair. Histological staining revealed that the structure of the native hyaline cartilage is preserved and a thin layer of subchondral bone is present within the CVOCA. The CVOCA retains and secretes ECM proteins and growth factors that are typically found in hyaline cartilage. The CVOCA was non-immunogenic and contained viable chondrocytes that stained positive for aggrecan and type II collagen. Functionally, the CVOCA induces MSC migration and triggers MSC chondrogenesis. The highest level of chondrogenesis *in vitro* was seen when the MSCs were cultured within the pores of the CVOCA. These results support the hypothesis that the CVOCA can induce chondrogenesis of MSCs recruited from the bone marrow and trapped within the CVOCA pores following marrow stimulation, producing type II collagen and restoring damaged cartilage.

Including flash frozen OCAs, which do not contain viable chondrocytes, in some of our experiments revealed the importance of preserving all components of the CVOCA (ECM, growth factors, cells). The flash frozen OCA showed decreased growth factor release, MSC migration, and MSC chondrogenesis relative to the CVOCA with preserved living cells. These results indicate that viable chondrocytes continue to secrete growth factors, contributing to the persistence of growth factor secretion over time from the CVOCA and the resulting increase in MSC migration and chondrogenesis. The high percentage of viable cells preserved within the CVOCA (70.5 %) is a significant improvement compared to previous attempts at cartilage cryopreservation that resulted in only 20–50 % viable cells and, due to the known importance of viable cells for graft performance, prevented cryopreserved osteochondral allografts from gaining widespread use in the past [35–37].

The *in vivo* goat study further supports our hypothesis that the CVOCA improves repair compared to marrow stimulation alone. Defect filling and gross tissue appearance of lesions treated with marrow stimulation augmented with the CVOCA were superior (significantly higher normalized grade scores) to lesions treated with marrow stimulation alone at 3 months, suggesting that the CVOCA facilitates a faster repair than marrow stimulation alone. Histologically, the pores of the CVOCA were completely filled with highly cellular, type II collagen and proteoglycan-rich repair tissue at 3 months. By 12 months, gross and histological analyses showed integration of the implanted CVOCA with the host cartilage and bone. The goats treated with marrow stimulation alone had less lesion fill and zonal organization within the repair tissue. The study also demonstrated that the preferred fixation technique of the CVOCA is to suture or apply fibrin glue around the periphery or over the top of the CVOCA rather than at the base of the lesion. We hypothesize that the fibrin glue beneath the CVOCA reduces infiltration of host MSCs and inhibits integration with the subchondral bone and should therefore be avoided.

Our *in vitro* and *in vivo* data are validated by a recent clinical case [38]. In this case study, the first documenting clinical use of the CVOCA, Hoffman et al. described a biopsy that was collected 9 months following implantation of the CVOCA in a 10-mm diameter trochlear defect. Histological staining of the biopsy showed 85 % hyaline cartilage repair tissue with strong type II collagen and proteoglycan staining, good cellularity, and a replicated tidemark. The pores of the CVOCA appeared filled upon inspection, and the patient reported resolution of pain and return to normal activity.

Clinically, the CVOCA has been used both as an adjunct to marrow stimulation and as a primary repair without deep marrow stimulation, more similar to a traditional osteochondral allograft. In the latter case, debridement of the cartilage and subchondral bone is performed until a bleeding bed is created within the lesion. The bleeding bed results in the formation of a blood clot that may help with integration of the implanted CVOCA. It is hypothesized that cartilage repair with the CVOCA will be mediated by the same mechanisms known for fresh stored osteochondral allografts. The CVOCA has a thin layer of bone which assists in integration with the patient’s own subchondral bone. However, the bone portion in the CVOCA was further reduced compared to fresh stored OCAs to enable the CVOCA to be flexible and to minimize damage to a patient’s healthy subchondral bone. Additional benefits to the CVOCA compared to fresh stored osteochondral allografts include a longer shelf life and no requirement for donor matching.

## Conclusions

A novel cryopreserved viable osteochondral allograft (CVOCA), with pores and a reduced bone portion compared with a traditional osteochondral allograft, has been developed. The CVOCA maintains the structure and contents of native hyaline cartilage (ECM, growth factors, chondrocytes). This CVOCA induces MSC migration and chondrogenesis that can improve repair following a bone marrow stimulation procedure. Unique attributes of the CVOCA include its flexibility to fit defects of any shape and contour, a straightforward implantation technique, and a long shelf life that enables point of care use within a single surgery.
